# Characterising skull pathologies in the *Sost*-deficient mouse for preclinical evaluation of sclerosteosis treatments

**DOI:** 10.1242/dmm.052839

**Published:** 2026-08-26

**Authors:** Timothy J. Dreyer, Eleanor Gouldie, Jacob A. C. Keen, Phil Salmon, Gill Holdsworth, Andrew A. Pitsillides, Scott J. Roberts

**Affiliations:** ^1^ Skeletal Biology Group, Department of Comparative Biomedical Sciences, The Royal Veterinary College, London NW1 0TU, UK; ^2^ Bruker, 2550 Kontich, Belgium; ^3^ UCB Pharma, Slough SL1 3WE, UK

**Keywords:** Sclerosteosis model, Skull, Ossicle, Mandible

## Abstract

Facial paralysis, hearing loss and potentially lethal raised intracranial pressure are primary symptoms of sclerosteosis, an ultra-rare, autosomal recessive high bone mass (HBM) condition that predominantly manifests in skull pathologies. Non-invasive treatment remains an unmet clinical need. Recently, PORCN inhibition has been shown to reduce bone mass in young Sost-deficient (*Sost*^−/−^) mice. In this paper, we characterised sclerosteosis skull pathologies at later adult stages using a *Sost*^−/−^ mouse model, with a view to drug efficacy evaluation. This revealed that male and female 9-month-old *Sost*^−/−^ mice recapitulate the skull overgrowth and neural impingement of human sclerosteosis. We observed that the foramen magnum was narrowed in *Sost*^−/−^ mice, and that parietal bone mass and thickness were significantly elevated. Otosclerosis was apparent, and animals exhibited significant hearing loss at age 8-9 months. Further, prominent mandibles highlighted hyperostosis in the jaw. Sexual dimorphism was also evident in the *Sost*^−/−^ mice, underscoring the importance of including both sexes in HBM studies. This study provides new insight into *Sost*^−/−^-skull-related pathology and further validates *Sost*^−/−^ mice as a valuable model for investigating potential treatments for sclerosteosis and other HBM related conditions.

## Teaser

**Summary:** This study provides new insight into the skull pathology of *Sost*^−/−^ mice, further validating *Sost*^−/−^ mice as a valuable model for investigating potential treatments for sclerosteosis and other high-bone-mass-related conditions.

## INTRODUCTION

Sclerosteosis [Online Mendelian Inheritance in Man (OMIM) #269500] is an autosomal recessive sclerosing bone dysplasia marked by progressive skeletal overgrowth, especially of the skull and mandible (Beighton et al., 1976). This ultra-rare, extreme high bone mass (HBM) condition occurs worldwide (with <100 cases identified between 1896 and 2011), but manifests predominantly in the South African Afrikaner population (van Lierop et al., 2017). Sclerosteosis is caused by nonsense, missense or frameshift mutations in the gene encoding sclerostin (*SOST*), a secreted glycoprotein, resulting in altered expression and processing of the protein (Brunkow et al., 2001; Balemans et al., 2001, 2002; Poole et al., 2005). Sclerostin is primarily secreted by osteocytes and act as a potent negative regulator of bone formation, mainly through local control of the canonical Wnt/β-catenin signalling pathway (Lin et al., 2009; Nusse and Clevers, 2017; Holdsworth et al., 2019). Although knowledge of the mechanisms that control the emergence of HBM in sclerosteosis has helped in developing a novel sclerostin antibody therapy for osteoporotic individuals, treatment to restrict sclerosteosis-related pathologies is not yet available (Brunkow et al., 2001; Mullard, 2019).

The predominant clinical manifestations of sclerosteosis stem from excessive and dense cranial bone development. This leads to restricted intracranial volume and subsequent raised intracranial pressure (ICP), the primary threat to life. Together with a narrowed foramen magnum, raised ICP may cause brainstem compression, herniation and sudden death (Stein et al., 1983; du Plessis, 1993; Hamersma et al., 2003). Hyperostosis and narrowing of the jugular canal can also compress the carotid artery and jugular vein, producing intracranial hypertension, whilst cervical syrinx may result from cerebrospinal fluid flow disruption around the spinal cord or lower brain stem, thus, interfering with neuronal impulse transmission (Stein et al., 1983; du Plessis, 1993). Less debilitating contraction of the external auditory canal and middle ear cause progressive conductive hearing loss from early childhood onwards (Nager and Hamersma, 1986; Hamersma and Hofmeyr, 2007). Hearing loss is exacerbated by excess bone impinging on the ossicles and oval/round cochlear windows (Hamersma and Hofmeyr, 2007; Potgieter et al., 2014).

Foraminal narrowing by bone encroachment also entraps cranial nerves, causing facial palsy, sensorineural hearing loss (vestibulocochlear nerve) and the potential for later vision loss (optic nerve), neuralgia (trigeminal nerve) and anosmia (olfactory nerve) (Beighton et al., 1976; Cremin, 1979; Nager and Hamersma, 1986; Hamersma and Hofmeyr, 2007; de Andrade et al., 2013). Pronounced facial deformities, including mandibular overgrowth and forehead bossing, typically emerge during childhood and progress in adulthood but are unlikely to threaten life (Beighton and Hamersma, 1979; Cremin, 1979; van Lierop et al., 2017). Asymmetrical mandibular hypertrophy with mandibular and palatal tori and muscle weakness due to facial nerve paralysis may, together with anodontia and/or delayed tooth eruption, also hinder mastication (Stephen et al., 2001). These pathologies underlie the morbidity and mortality associated with sclerosteosis, and complicate the high-risk alleviating surgeries required to manage the condition, highlighting the need for an alternative therapy for this rare condition (du Plessis, 1993; Stephen et al., 2001; van Lierop et al., 2017; Dreyer et al., 2021, 2025; Appelman-Dijkstra et al., 2024).

Sost-deficient (*Sost*^−/−^) and transgenic *Sost*-overexpressing mouse models have been pivotal in elucidating the role of sclerostin in bone biology, resulting in subsequent development of anti-sclerostin therapeutics (Winkler et al., 2003; Loots et al., 2005; Li et al., 2008). Li et al. (2008) used *Sost* gene deletion (C57BL/6 background) to develop a *Sost* knockout (KO) model. They characterised serum markers and femoral/vertebral bone parameters in 4- to 6.5-month-old male and female KO mice and found an HBM phenotype akin to human sclerosteosis. However, unlike humans, *Sost*^−/−^ mice had no signs of facial distortion or paralysis and no greater mortality at this age. Despite such extensive characterisation of the axial and appendicular skeleton of *Sost*^−/−^ mice, few studies have examined the primary site of sclerosteosis pathology, the skull.

Although Sost deficiency produces analogous phenotypic bone modifications in male and female mice, some parameters exhibit sexual dimorphism in magnitude (Li et al., 2008). Indeed, female *Sost*^−/−^ mice exhibit a greater load-induced osteogenic response, indicating that sclerostin exerts distinct sexually dimorphic control of cortical bone responses to loading (Pflanz et al., 2017; Albiol et al., 2020; Yang et al., 2020). Furthermore, sex-related differences in bone mass and/or the anabolic response to mechanical loading in mice with altered Wnt/β-catenin signalling have been revealed in several studies (Sawakami et al., 2006; Kramer et al., 2010; Saxon et al., 2011; Javaheri et al., 2012, 2014). This dimorphism highlights the need to include male and female animals in studies evaluating therapies for bone-related diseases, and in considering sex in preclinical studies, to fully capture treatment responses (Sharma et al., 2023). Indeed, we and others have recently shown that PORCN inhibition modifies vertebral and long bone parameters of young *Sost*^−/−^ mice, with our work also extending to effects on the skull (Diegel et al., 2023; Dreyer at al. 2025). However, changes were more pronounced in male than female mice, indicating that sexual dimorphism should be considered if this model is used for therapeutic discovery.

Here, we characterise the skulls of  9-month-old male and female *Sost^−/−^* mice to complement existing data on the appendicular and axial skeleton (Li et al., 2008). The rationale for choosing 9 months as the study end point is due to this stage being classed as middle aged (∼35-40 years old) in human terms, by which time all sclerosteosis pathologies are present in humans (Flurkey et al., 2007; Appelman-Dijkstra et al., 2024). Moreover, previous studies have shown that BMD levels continued to increase until 40 weeks of age in *Sost*^−/−^ mice (Lin et al., 2009; Ke et al., 2012). Cranial morphology, including parietal thickness, intracranial volume, foramen magnum size, mandibular and middle/inner ear ossicle size as well as auditory function were assessed to define whether *Sost* deficiency in mice recapitulates the skull overgrowth and progressive nerve impingement seen in human sclerosteosis patients; evaluation in both sexes addressed any potential sexual dimorphism. By characterising skeletal and sensory phenotypes, this study defines *Sost*^−/−^ mice as a model for sclerosteosis-like pathology and that these mice are suitable for the evaluation of new potential treatments for *SOST*-related HBM disorders.

## RESULTS

### Increased skull bone mass and parietal bone thickness in *Sost*^−/−^ mice

**Fig. 1.**
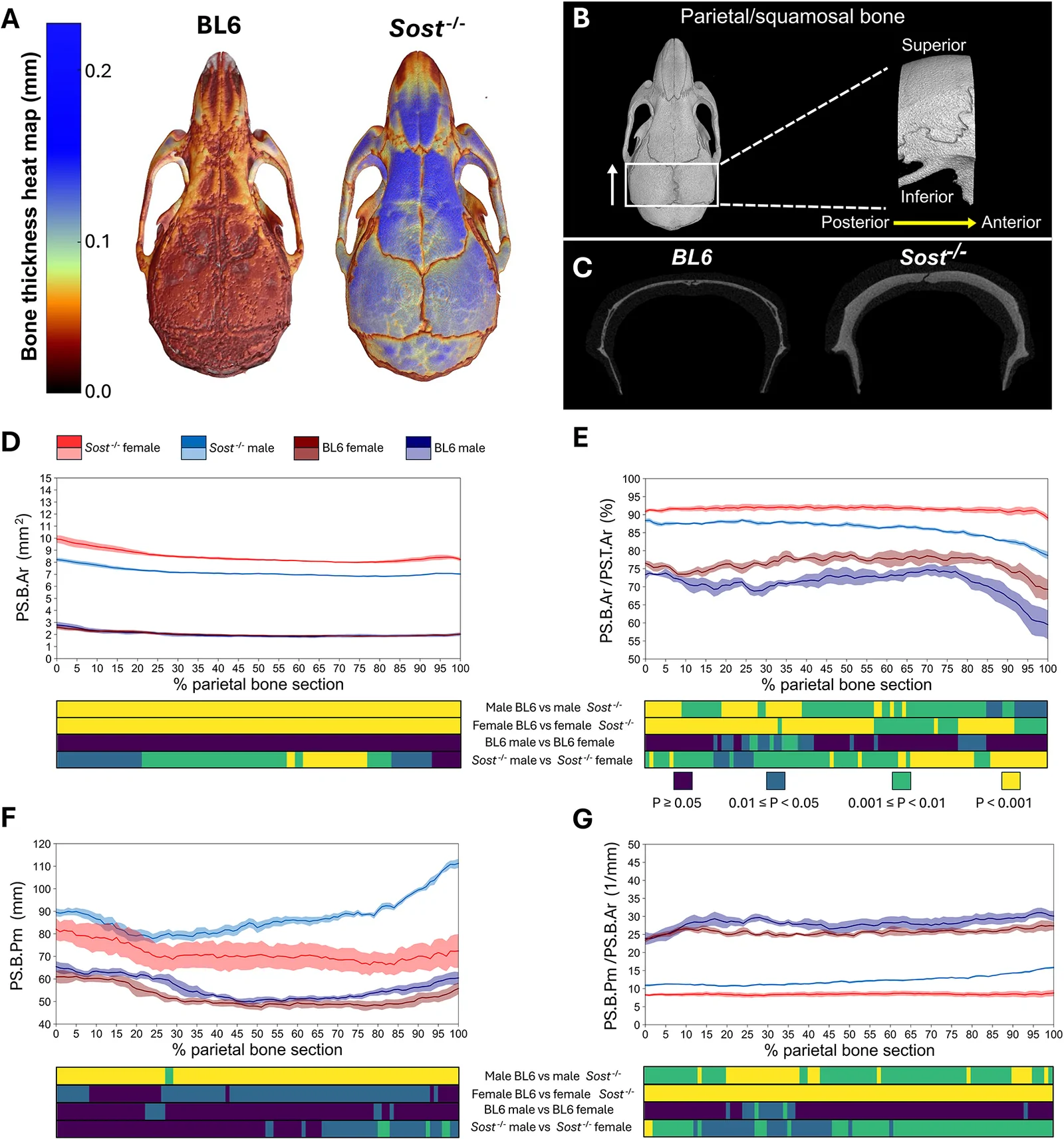
**Increased thickness and hyperostosis of parietal/squamosal bone in male and female** ***Sost***^**−/−**^ **mice.** (A) Representative 3D microCT images of skulls (top view) from BL6 and *Sost*^−/−^ female mice aged 9 months, showing their respective colour-coded bone thickness. (B) Representative top-view image of the skull from a female *Sost*^−/−^ mouse. The boxed area indicates the analysed region between anterior lambdoid and coronal sutures (shown magnified on the right; lateral view). Whole-bone analysis was done from anterior to posterior (yellow arrow) of the selected region. (C) Representative trans-axial cross-section images of the parietal bone from female BL6 and *Sost*^−/−^ mice as indicated. (D-G) Line graphs (mean±s.e.m) showing increases in the parietal/squamosal bone area (PS.B.Ar) (D), bone area percentage (PS.B.Ar/PS.T.Ar) (E) and bone perimeter (PS.B.Pm) (F), and a decrease in bone perimeter/bone area fraction (PS.B.Pm/PS.B.Ar) (G) of the parietal/squamosal bone region in 9-month-old male and female *Sost*^−/−^ mice compared to BL6 counterparts [BL6 males (dark blue); *Sost*^−/−^ males (light blue); BL6 females (maroon); *Sost*^−/−^ females (red)]. Sexual dimorphism is evident in D-G for *Sost*^−/−^ mice. Heat maps below graphs summarise statistical differences of the above groups at matched locations along the analysed parietal bone length (dark blue, *P*≥0.05; blue, 0.01≤*P*<0.05; green, 0.001≤*P*<0.01; yellow, *P*<0.001); *n*=4 BL6 males, *n*=4 BL6 females, *n*=6 *Sost*^−/−^ males and *n*=4 *Sost*^−/−^ females. A linear mixed-effects model was used to assess significant differences between male and female BL6 and *Sost*^−/−^ mice at each distinct percentile along the bone section length. Shapiro–Wilk tests were performed to assess normality of residuals, and normality was assumed if most measures (10-90%) along the parietal bone section satisfied the null hypothesis of normality.

Increased bone mass in the skulls of 9-month-old *Sost*^−/−^ mice replicates the predominant sclerosteosis-related pathology (Fig. 1A). In sclerosteosis patients, potentially fatal raised ICP is caused by a decrease in cranial volume resulting from an increase in parietal and frontal bone thickness. As such, 9-month-old *Sost*^−/−^ mouse parietal/squamosal bone morphometric parameters were measured (from posterior to anterior) to investigate potential skull differences (Fig. 1B). *Sost*^−/−^ bone was thicker than that in C57BL6 wild-type control mice (hereafter referred to as BL6) across the whole parietal/squamosal bone region (Fig. 1C; Fig. S1A,H; Table S1). Bone mass was also significantly greater, with parietal/squamosal bone volume (PS.BV) and total volume (PS.TV) increased (∼2-fold, *P*≤0.0001) in *Sost*^−/−^ mice (Fig. 1A; Fig. S1B,C; Table S1). Furthermore, *Sost*^−/−^ mice had a higher parietal/squamosal bone volume fraction (PS.BV/PS.TV) and bone surface (PS.BS), while specific bone surface (PS.BS/PS.BV) was lower (Fig. S1D-F; Table S1). These differences in mass and other morphometric parameters were evident across the entire examined portion of parietal/squamosal bone (Fig. 1D-G; Fig. S1H-J). *Sost*^−/−^ parietal/squamosal bone had larger total (PS.T.Ar) and bone (PS.B.Ar) areas, accompanied by a higher bone fraction (PS.B.Ar/PS.T.Ar) and longer bone perimeter (PS.B.Pm) (Fig. 1D-F). Upon comparison of BL6 versus *Sost*^−/−^ mice, lower parietal/squamosal bone perimeter/area fraction (PS.B.Pm/PS.B.Ar), PS.BS/PS.BV (male: −53%; female: −64%; *P*≤0.0001) and bone porosity (PS bone porosity; male: −53%; female −65%; *P*≤0.0001) were evident in *Sost*^−/−^ mice, highlighting significantly less porous bone (Fig. 1G; Fig. S1G; Table S1).

Notably, this increased mass and parietal/squamosal bone thickness only decreased total intracranial volume in female 9-month-old Sost-deficient mice (Table S1). Sexual dimorphism was further evident in *Sost*^−/−^ mice, with higher average parietal/squamosal bone mass and volume in females compared to males (bone thickness: 34%; *P*≤0.001; PS.BV: 28%; *P*≤0.0001; PS.TV: 21%; *P*≤0.0001; PS.BV/PS.TV: 7%; *P*≤0.01), whilst PS.BS remained similar (Fig. 1D,E; Fig. S1A-E; Table S1). Interestingly, bone in male *Sost*^−/−^ mice was more porous than in female *Sost*^−/−^ mice, with higher PS.B.Pm/PS.B.Ar (across the whole section), PS.BS/PS.BV (31%; *P*≤0.01) and PS bone porosity (40%; *P*≤0.01) (Fig. 1G; Fig. S1F,G; Table S1).

### Narrowed foramen magnum in *Sost*^−/−^ mice

**Fig. 2.**
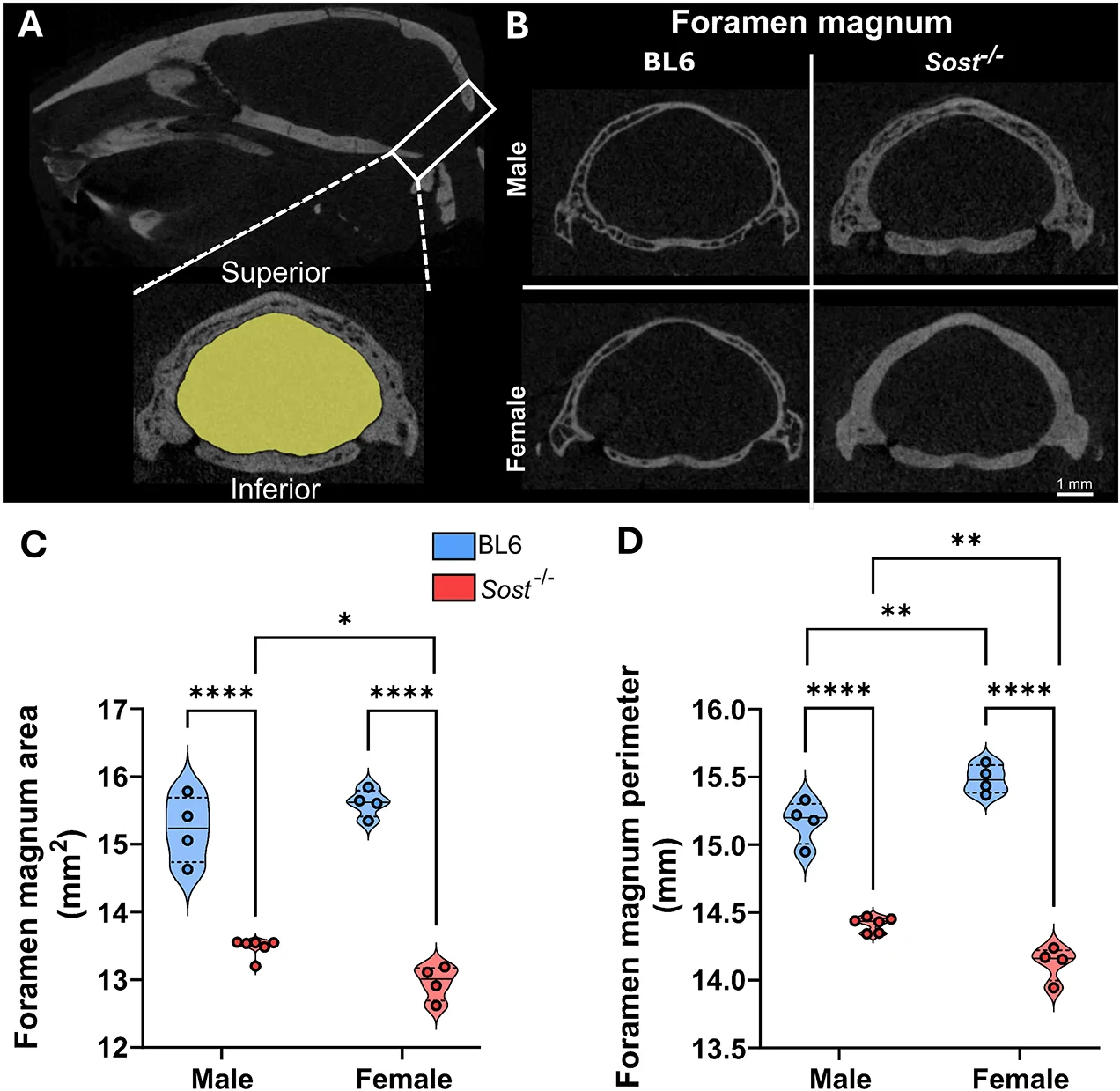
**Increased bone thickness and narrowed foramen magnum in male and female** ***Sost***^**−/−**^ **mice.** (A) Top: medial cross-section of a 9-month-old *Sost*^−/−^ mouse skull; the boxed area indicates the location the foramen magnum. Bottom: magnified image of the foramen magnum (boxed area in top image), with the measured area highlighted in yellow. (B) Representative cross-sectional 2D microCT images of the foramina magna of male (top) and female (bottom) BL6 (left) and *Sost*^−/−^ (right) mice aged 9 months. (C,D) Violin plots showing the decreased foramen magnum area (C) and the perimeter (D) of the *Sost*^−/−^ mice compared to BL6 counterparts. Sexual dimorphism is evident for both BL6 and *Sost*^−/−^ mice. All plots: the mean is indicated by a solid line, and upper and lower dashed lines represent quartiles. *P≤0.05, **P≤0.01, ****P≤0.0001; *n*=4 BL6 males, *n*=4 BL6 females, *n*=6 *Sost*^−/−^ males, *n*=4 *Sost*^−/−^ females. Two-way ANOVA with Šidák's multiple comparison tests was used to determine statistically significant differences for two-group comparisons.

Narrowing of the foramen magnum contributes to potential pressure on the vertebral arteries, raised-ICP-related brain coning and sudden death in sclerosteosis patients. The area of the foramen magnum was, therefore, measured in sex-matched 9-month-old mice (Fig. 2A,B), which revealed narrowed foramina magna in *Sost*^−/−^ mice, with decreased foramen area (male: −11%; female: −17%; *P*≤0.0001) and perimeter (male: −5%; female: −9%; *P*≤0.0001) in both sexes (Fig. 2C,D; Table S1). Although not analysed, bone around the foramen magnum was visibly more porous in male than in female *Sost*^−/−^ mice (Fig. 2B). Sexual dimorphism was further highlighted by a narrower foramen magnum in female *Sost*^−/−^ mice, with a shorter perimeter (−4%; *P*≤0.05) and smaller area (−2%; *P*≤0.01) compared to those of males (Fig. 2C,D; Table S1). In contrast to *Sost*^−/−^ mice, the foramen magnum perimeter in 9-month-old female BL6 mice was moderately increased (2%; *P*≤0.01) compared with that of males, whilst the foramen magnum area remained equivalent (Fig. 2C,D; Table S1).

### Increased middle/inner ear bone mass is coupled to hearing loss in *Sost*^−/−^ mice

**Fig. 3.**
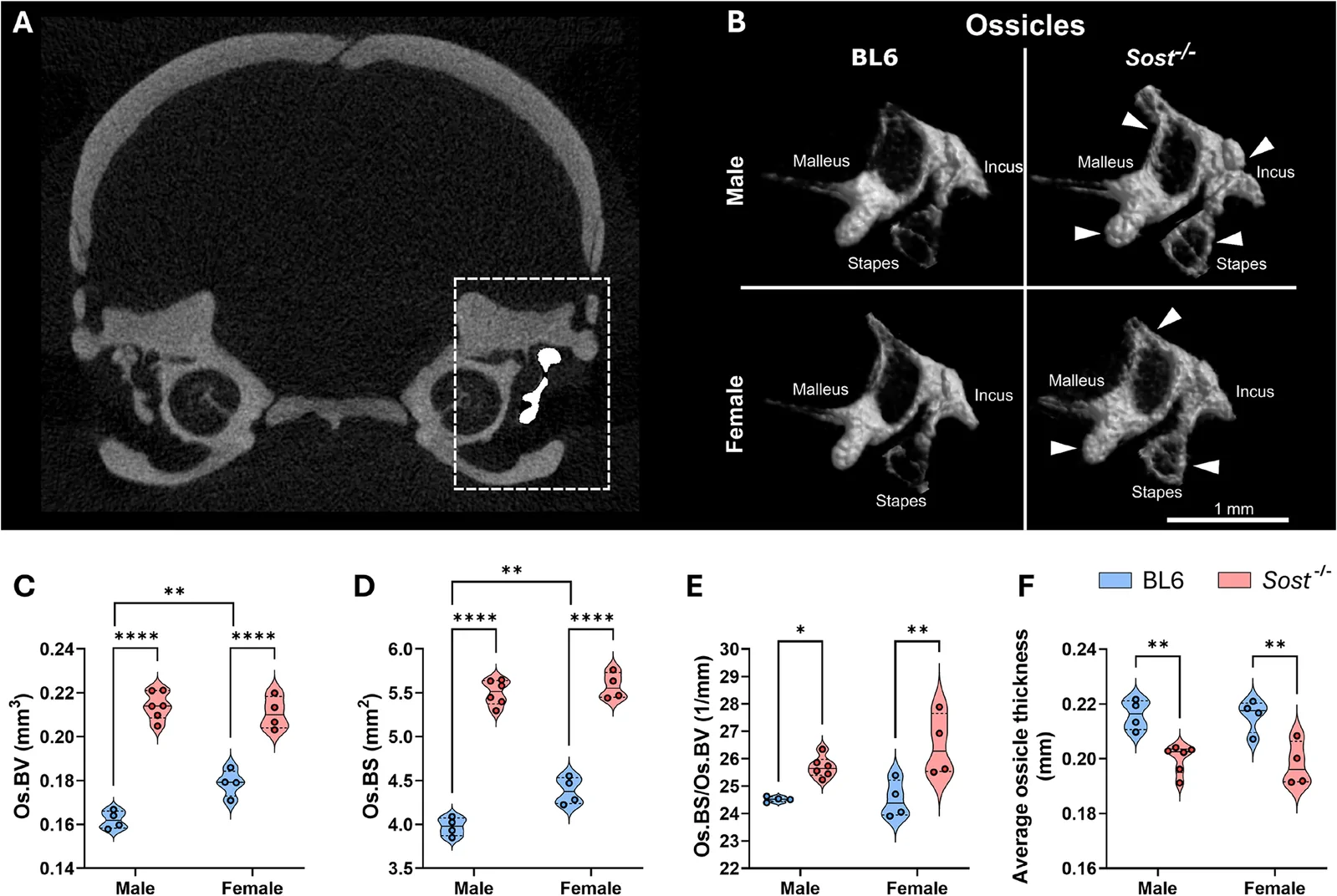
**Otosclerosis in the middle ear of** ***Sost***^**−/−**^ **mice.** (A) Image showing a trans-axial cross-section of a 9-month-old female *Sost*^−/−^ mouse skull; the ossicles region of interest is highlighted in white within the boxed area. (B) Representative 3D microCT images of the ossicles in the middle ear of male (top) and female (bottom) BL6 (left) and *Sost*^−/−^ (right) mice aged 9 months. Arrowheads indicate regions with a visible increase in bone. (C-F) Violin plots showing the increased ossicle bone volume (Os.BV) (C), ossicle bone surface (Os.BS) (D) and ossicle bone surface fraction (Os.BS/Os.BV) (E), and decreased average ossicle thickness (F) in *Sost*^−/−^ mice compared to their BL6 counterparts, as indicated. Sexual dimorphism is evident for BL6 mice. All plots: the mean is indicated by solid lines; upper and lower dashed lines represent quartiles. *P≤0.05, **P≤0.01, ****P≤0.0001; *n*=4 BL6 males, *n*=4 BL6 females, *n*=6 *Sost*^−/−^ males, *n*=4 *Sost*^−/−^ females. Two-way ANOVA with Šidák's multiple comparison tests was used to determine statistically significant differences for two-group comparisons.

Given that sclerosteosis patients suffer from HBM-linked conductive and sensorineural hearing loss, we examined whether the *Sost*^−/−^ mouse exhibits similar differences in middle and inner ear structure and hearing capacity. Comparison across both sexes revealed otosclerosis and increased ossicle bone volume (Os.BV; male: 32%; female: 18%; *P*≤0.0001), surface (Os.BS; male: 38%; female: 27%; *P*≤0.0001) and surface density (Os.BS/Os.BV; male: 5%; *P*≤0.05 and female: 8%; *P*≤0.01) in the middle ear of 9-month-old *Sost*^−/−^ compared with that of 9-month-old BL6 mice (Fig. 3A-E; Table S1). Interestingly, although bone mass and surface of BL6 mice were reduced compared with those of their *Sost*^−/−^ counterparts, BL6 ossicles exhibited 7% and 8% (*P*≤0.01) increased average thickness in male and female, respectively (Fig. 3F; Table S1). Whilst bone morphometric parameters were similar in *Sost*^−/−^ male and female ossicles, female BL6 mice exhibited a 10% (*P*≤0.01) increase in Os.BV and Os.BS compared with BL6 males.

**Fig. 4.**
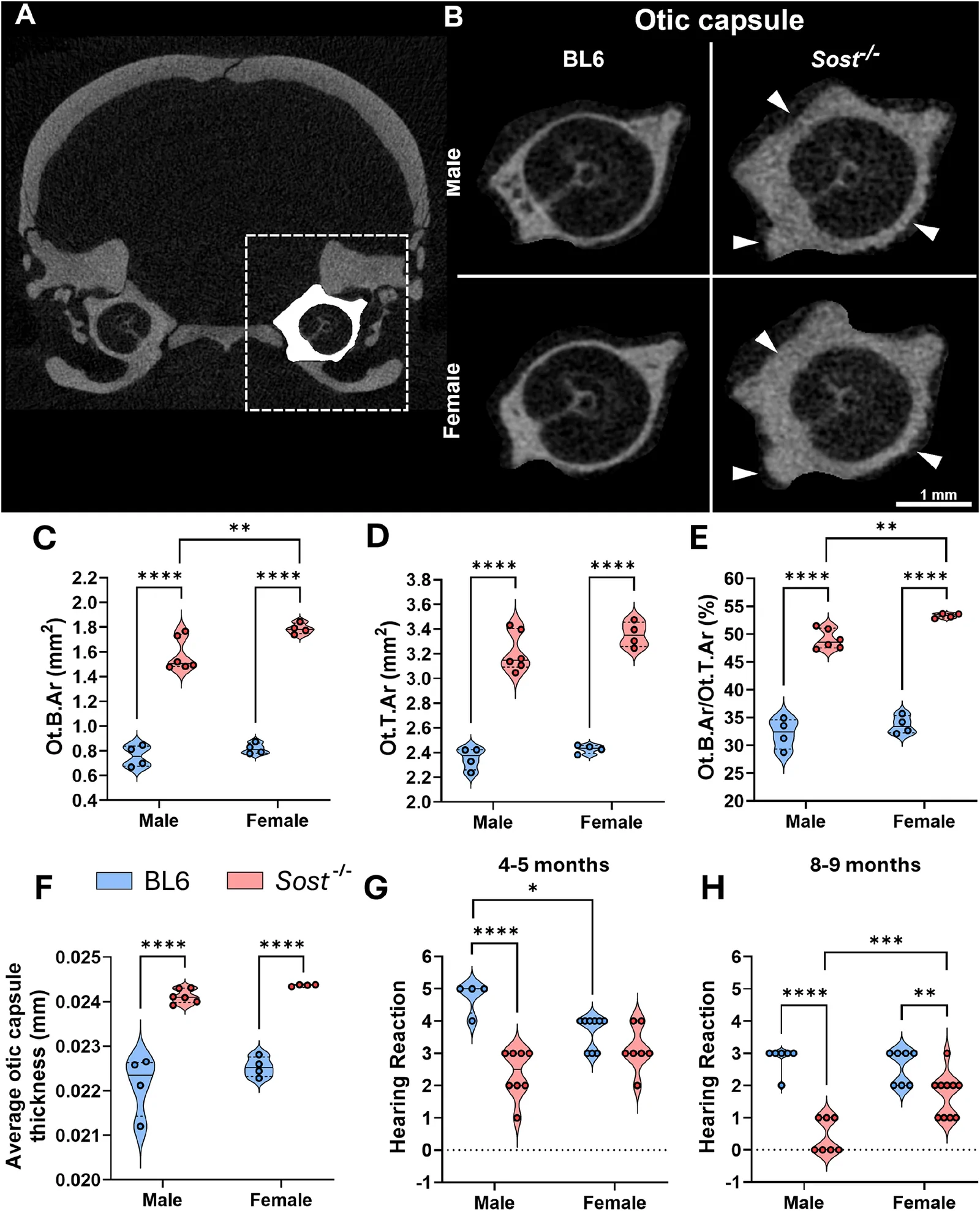
**Increase in inner ear bone mass is accompanied by hearing loss in** ***Sost***^**−/−**^ **mice.** (A) Image showing a trans-axial cross-section of a 9-month-old female *Sost*^−/−^ mouse skull; the otic capsule is highlighted in white within the boxed area. (B) Representative cross-sectional 2D microCT images of inner ear capsules obtained from male (top) and female (bottom) BL6 (left) and *Sost*^−/−^ (right) mice. Arrowheads indicate regions with a visible increase in bone. (C-F) Violin plots showing increases in the area of the otic capsule cross-sectional bone (Ot.B.Ar) (C) and total area (Ot.T.Ar) (D), the percent bone area fraction (Ot.B.Ar/Ot.T.Ar) (E) and average bone thickness (F) in male and female *Sost*^−/−^ mice compared to their BL6 counterparts. (G,H) Violin plots showing the hearing loss in male and female BL6 and *Sost*^−/−^ mice aged 4-5 months (G) and 8-9 months (H) as indicated. Sexual dimorphism is evident for both BL6 and *Sost*^−/−^ mice. All plots: the mean is indicated by solid lines; upper and lower dashed lines represent quartiles (obstructed by data points in G and H). *P≤0.05, **P≤0.01, ***P≤0.001, ****P≤0.0001. For morphometric parameters, *n*=4 BL6 males, *n*=4 BL6 females, *n*=6 *Sost*^−/−^ males and *n*=4 *Sost*^−/−^ females. Hearing reaction in BL6 and *Sost*^−/−^ age and sex groups varied in size, with *n*≥4. Two-way ANOVA with Šidák's multiple comparison tests was used to determine statistically significant differences for two-group comparisons.

The otic capsule bone area (Ot.B.Ar) in the inner ear of both male and female 9-month-old *Sost*^−/−^ mice was more than double (1.58 mm^2^ versus 0.76 mm^2^ and 1.79 mm^2^ versus 0.82 mm^2^ for males and females, respectively; *P*≤0.0001) that of BL6 mice (Fig. 4A-C; Table S1). Total otic capsule area (Ot.T.Ar) and bone fraction (Ot.B.Ar/Ot.T.Ar) were also increased in *Sost*^−/−^ mice (Fig. 4D,E; Table S1) and capsules were ∼8% thicker (*P*≤0.01 and *P*≤0.0001 for male and female, respectively) than BL6 (Fig. 4B,F; Table S1). Sexual dimorphism was apparent, with increased Ot.B.Ar (13%; *P*≤0.01) and Ot.B.Ar/Ot.T.Ar (9%; *P*≤0.01) in female compared to male *Sost*^−/−^ mice (Fig. 4C,E; Table S1).

As hearing loss is a prominent sclerosteosis symptom, response to audio stimuli was assessed using a non-invasive method to determine hearing capacity during the lifetime of the mouse. At age 4-5 months, male *Sost*^−/−^ mice exhibited a 50% reduction (*P*≤0.0001) in the response to audio stimuli compared to their BL6 counterparts (Fig. 4G; Table S1). Furthermore, responsiveness to audio stimuli of younger (4- to 5-month-old) male BL6 mice was better (23%, *P*≤0.05) than that of female BL6 mice of that age. Hearing significantly declined by 85% (*P*≤0.0001) and 34% (*P*≤0.01) in older (9-month-old) *Sost*^−/−^ males and females, respectively, with male *Sost*^−/−^ mice exhibiting a notably increased degree of hearing loss (∼300%, *P*≤0.001) than females (Fig. 4H; Table S1).

### Hyperostosis in the mandible of *Sost*^−/−^ mice

**Fig. 5.**
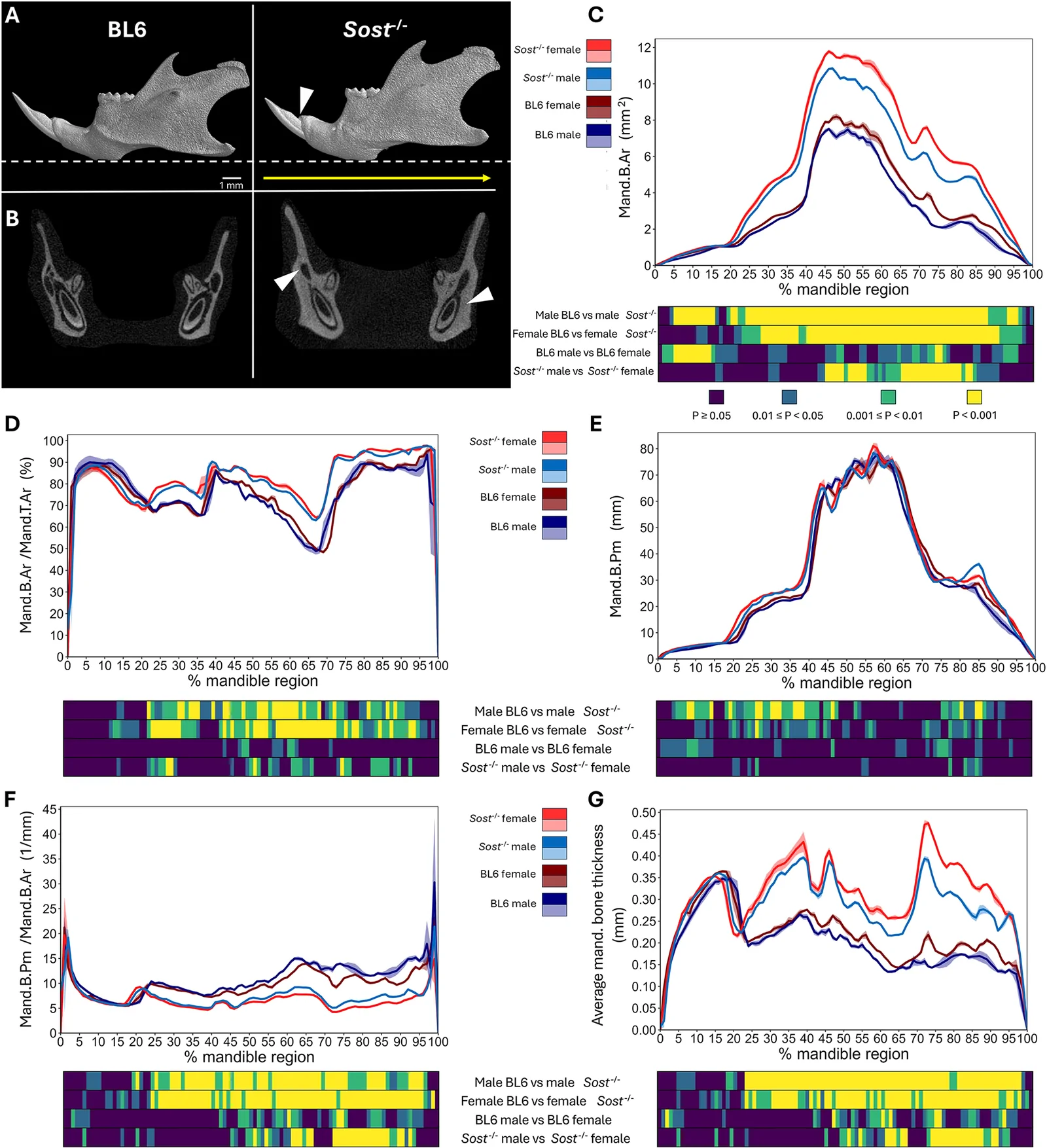
**Mandibular hyperostosis in male and female** ***Sost***^**−/−**^ **mice.** (A) Representative images of 9-month-old female BL6 (left) and *Sost*^−/−^ (right) mouse mandibles, highlighting bone overgrowth around the mandibular canal and incisor base (white arrowheads) in the *Sost*^−/−^ mouse. Whole-bone analysis was done anterior to posterior (yellow arrow) of the selected region. BL6 and *Sost*^−/−^ mandibles were aligned based on most ventral and rostral point of the insertion area of the digastric muscle. (B) Representative trans-axial cross-section images of the mandible in female BL6 (left) and *Sost*^−/−^ (right) mice aged 9 months, highlighting bone overgrowth around the mandibular canal and incisor base (white arrowheads) in the *Sost*^−/−^ mouse. (C-G) Line graphs (mean±s.e.m) showing the increased bone area (Mand.B.Ar) (C), percent bone area (Mand.B.Ar/Mand.T.Ar) (D) and bone perimeter (Mand.B.Pm) (E), decreased bone perimeter to bone area ratio (Mand.B.Pm/Mand.B.Ar (F) and increased average bone thickness (G) of male and female 9-month-old *Sost*^−/−^ mandibles compared to BL6. BL6 males (dark blue); *Sost*^−/−^ males (light blue); BL6 females (maroon); *Sost*^−/−^ females (red). Heat maps below the graphs summarise statistical differences at specific matched locations along the analysed length of the parietal bone section (dark blue, *P*≥0.05; blue, 0.01≤*P*<0.05; green, 0.001≤*P*<0.01; yellow *P*<0.001). *n*=4 BL6 males, *n*=4 BL6 females, *n*=6 *Sost*^−/−^ males, *n*=4 *Sost*^−/−^ females. A linear mixed-effects model was used to assess significant differences between male and female BL6 and *Sost*^−/−^ mice at each distinct percentile along the bone section length. Shapiro–Wilk tests were performed to assess normality of residuals, and normality was assumed if most measures (10-90 %) along the parietal bone section satisfied the null hypothesis of normality.

Mandibular hyperostosis in sclerosteosis patients restricts gape (the ability to open the mouth), further complicating already challenging surgeries. As in human patients, 9-month-old *Sost*^−/−^ mice exhibit prominent mandibular hyperostosis (Fig. 5A,B; Fig. S2A), with bone encroachment of the incisor root and mandibular canal (visible in the trans-axial view). Furthermore, some narrowing of the mental foramen (−22%; *P*≤0.05) in male *Sost*^−/−^ mice was evident compared to male BL6 controls (Fig. 5B; Fig. S2A,B; Table S1). Distances between mandibular landmarks further highlighted the larger nature of *Sost*^−/−^ mandibles (Fig. S3). Incisors in *Sost*^−/−^ mice appeared shorter due to bone encroachment and increased mandibular bone mass (Fig. 5A,B; Fig. S3A,B). Furthermore, the mandibles of *Sost*^−/−^ mice were thicker than sex- and age-matched BL6 control mice, with significant mandibular hyperostosis across the mandible length in both sexes (Fig. 5C-G; Fig. S2C,D; Fig. S4A; Table S1). These findings were supported by increases in mandibular bone volume (Mand.BV; >65%; *P*≤0.0001), total volume (Mand.TV; 45%; *P*≤0.0001), bone area percentage (Mand.BV/Mand.TV; 15%; *P*≤0.0001), bone surface (Mand.BS; 30%; *P*≤0.0001) and a decrease in bone surface fraction (Mand.BS/Mand.BV; −36%; *P*≤0.0001), accentuating a greater mandibular bone mass in both male and female *Sost*^−/−^ mice compared to BL6 (Fig. S4B,F; Table S1).

Sexual dimorphism was evident in the mandibles of 9-month-old *Sost*^−/−^ mice (and, incidentally, also in specific/alternate regions of the mandible of BL6 mice), with significantly higher bone mass in females than males (Fig. 5C,F,G). Consistent with comparisons across the length of the mandible, the female mandibular bone was less porous (Mand.BS/Mand.BV: −10%; *P*≤0.01), and had more mass and volume (Mand.BV: 13%; *P*≤0.0001; Mand.TV: 11%; *P*≤0.0001; Mand.BV/Mand.TV: 2%; *P*≤0.01; average mandibular bone thickness: 16%; *P*≤0.001) compared to *Sost*^−/−^ male mice (Fig. S4A-F; Table S1).

## DISCUSSION

This study demonstrates that a number of skull-associated sclerosteosis pathologies are recapitulated in the skulls of 9-month-old *Sost*^−/−^ mice. Our data show that the HBM phenotype – analogous to sclerosteosis in humans – is evident in the parietal bone, mandible and ear bones, with the latter linked to progressive hearing loss. Moreover, our findings reveal, for the first time, sexually dimorphic patterns of skeletal dysplasia in male and female *Sost*^−/−^ mouse skulls. Together, these findings demonstrate that *Sost*^−/−^ mice can serve as a meaningful model in which to evaluate the efficacy of new drugs to counteract the skeletal changes observed in human patients with this debilitating and life-threatening bone disease.

Previous studies on the post-cranial skeletons of *Sost*^−/−^ mice have reported a prominent HBM phenotype that resembles that observed in this ultra-rare human HBM condition, sclerosteosis (Li et al., 2008; Koide et al., 2022; Diegel et al., 2023). Focussed analyses highlighted that the complete lack of functional sclerostin in these mice resulted in elevated bone mass, and structural and mechanical changes in the femur, tibia and vertebrae. These studies have played a pivotal role in furthering our understanding of sclerostin function in bone modelling and remodelling (Li et al., 2008; Koide et al., 2022). Crucially, these previous studies provided the first evidence that *Sost*^−/−^ mice might also provide a model for investigating treatments for sclerosteosis, a debilitating HBM condition (Dreyer et al., 2021, 2025; Diegel et al., 2023).

However, these previous studies primarily focussed on the whole body, vertebrae and bones of the appendicular skeleton of *Sost*^−/−^ mice, omitting detailed evaluation of the skull, where the primary sclerosteosis symptoms – such as facial paralysis, hearing loss and raised ICP – occur. In this study, we have sought to address this considerable oversight, by examining in more detail the hyperostosis previously observed in various parts of the skull in the *Sost*^−/−^ mouse model (Dreyer et al., 2025). This involved investigating the bone phenotype in different regions of the skull, including the whole skull vault, parietal bone, foramen magnum, middle and inner ear bones, and the mandible, to gain a clearer understanding of how HBM affects their morphology within the skull of the *Sost*^−/−^ mice.

Our cursory observations of no apparent syndactyly, facial paralysis or diminished lifespan in *Sost*^−/−^ mice were consistent with previous studies (Li et al., 2008). This is, however, somewhat paradoxical and inconsistent in regard to human sclerosteosis symptoms – which present bony/cutaneous syndactyly, acute facial nerve palsy and shortened lifespan if left untreated (with a mean age of death at 33 years) – and it is assumed, but so far has not been addressed, that these differences are due to divergent human and mouse skeletal development, and anatomy.

We found that the total skull bone mass, as assessed by a wide range of different bone morphometric parameters, including thickness of the parietal bone, were significantly elevated in *Sost*^−/−^ mice, consistent with human patients (du Plessis, 1993). Narrowing of the foramen magnum also occurred in male and female *Sost*^−/−^ mice. However, in contrast to humans, whose raised ICP and narrowing of the foramen magnum may result in fatal impaction of the medulla oblongata, this pathology has not been seen/reported in mice (du Plessis, 1993; Hamersma et al., 2003). MRI-like brain scans would be required for further assessment of this pathology. Nonetheless, the significant scale of these changes in *Sost*^−/−^ mice provide a model in which quantifiable shifts towards a normal, healthy architecture of the occipital bone at the base of the skull can be measured to evaluate the efficacy of potential sclerosteosis drugs.

Conductive/sensorineural hearing loss caused by HBM ear bone changes is another prominent phenotype in sclerosteosis patients (Hamersma and Hofmeyr, 2007; Potgieter et al., 2014). Our current data align with our previous studies to reveal hyperostosis in the *Sost*^−/−^ middle and inner ear, resulting in otosclerosis, and increased ossicle and otic capsule bone mass (Dreyer et al., 2025). Interestingly, ossicles of BL6 mice were thicker than those of *Sost*^−/−^ mice; yet, ossicles of *Sost*^−/−^ mice had higher mass. This could be explained by flatter and fused *Sost*^−/−^ ossicles, structural changes that might increase bone mass and bone surface but result in a reduction of average bone thickness across the ossicles. We showed, for the first time, a decreased response to audio impulses in older *Sost*^−/−^ mice. Male hearing declined at an earlier age (4-5 months); but, by 9 months, both sexes exhibited a significant degree of hearing loss, with some mice showing no response to audio stimuli. Hearing loss in older and mature wild-type mice was less severe, indicating that the loss may be caused by impaired ossicle function and sound transmission through the oval window. Sensorineural hearing loss may also contribute to the lack of response to audio stimuli, although we were unable to measure any narrowing of the internal auditory canal, which could compress the vestibulocochlear nerve. It is an intriguing possibility that reversal of, or protection against, such hearing loss in *Sost*^−/−^ mice provides a non-invasive and accurate method for monitoring the efficacy of any potential sclerosteosis drug.

Enlarged mandibles are, likewise, a prominent feature in sclerosteosis patients, with the oversized mandible complicating already difficult surgeries by restricted gape (du Plessis, 1993; Stephen et al., 2001). Mandibular hyperostosis was evident in 9-month-old *Sost*^−/−^ mice, with significant increases in bone morphometric parameters. Moreover, there is some narrowing of the mental foramen in *Sost*^−/−^ males. As with humans, teeth in *Sost*^−/−^ mice can be malaligned due to mandibular hyperostosis (Appelman-Dijkstra et al., 2024). Indeed, research groups have shown that sclerostin plays an important role in bone remodelling of tooth movement and that the lack of sclerostin in *Sost*^−/−^ mice mainly alters the bone and cementum phenotypes rather than producing abnormalities in tooth structures, such as dentin in the molars (Kuchler et al., 2014; Shu et al., 2017).

Significant sexual dimorphism is evident in the emergent skull phenotype of *Sost*^−/−^ mice, with a more pronounced hyperostosis in females impacting the foramen magnum, parietal bone, mandible and the cochlea/otic capsule. Conversely, there was no clear difference in *Sost*^−/−^ female and male auditory ossicles, possibly due to conservation of ossicle architecture once fully formed during early stages of life (Rolvien et al., 2018). Notably, hearing loss onset occurred earlier and was more profound in males, despite females having higher ear bone mass (Turner et al., 1990). These sexual dimorphic differences might be attributed to physiological changes in aging males and females. Indeed, aged male and female BL6 mice experience a sharp drop in circulating oestrogen, a crucial negative regulator of bone resorption (from >20 pg/ml in 3- to 6-month-old mice to ≤1–5 pg/ml in 12- to 18-month-old mice), and testosterone, which plays a crucial role in bone homeostasis (from >8000 pg/ml in 3- to 4-month-old mice to <1000 pg/ml in 1-year-old mice) (Nelson et al., 1981; Vandenput et al., 2001; Syed et al., 2010; Sinnesael et al., 2012; Nilsson et al., 2015; Streicher et al., 2017). Therefore, sex-specific differences in the regulation of these hormones may contribute to observed variations in bone mass, demonstrating the need for further examination of the underlying basis for the observed sexual dimorphism in *Sost*^−/−^ mice. Interestingly, sexual dimorphic differences are also discernible in human sclerosteosis patients and heterozygous carriers when assessing hearing loss and Z-scores, even given the extremely low sample size (Tables S2, S3) (Gardner et al., 2005; Potgieter et al., 2014).

Although we gained novel insights into the *Sost*^−/−^ mouse model in this study, there are still other aspects of the *Sost*^−/−^ skull that should be explored. First, the type of hearing loss (conductive or sensorineural) in *Sost*^−/−^ mice should be elucidated to determine whether otosclerosis or nerve entrapment contributes most to hearing loss in these mice, possibly by including measurement of the auditory brainstem response. Second, measuring serum bone biomarkers and assessing gene expression in different regions of the skull could provide more information regarding the cause of sexual dimorphism in *Sost*^−/−^ mice. Indeed, an ovariectomised (OVX) or orchidectomised (ORX) *Sost*^−/−^ mouse study could provide valuable insights, increasing our understanding of sclerostin biology and sexual dimorphism, aiding the design of better treatments for *Sost*-related HBM conditions, such as sclerosteosis and Van Buchem disease (Wergedal et al., 2003). Third, although previously described by Li et al., immunolabelling, histology and histomorphometric analysis should be expanded to include skull bones of *Sost*^−/−^ mice to investigate potential differences in bone formation/resorption in different skeletal regions (Li et al., 2008).

Our findings provide new insight into the skull pathology of *Sost*^−/−^ mice and further highlight this mouse model as a crucial one for studying sclerosteosis. Further, we detail parameters that could be measured to aid the development of much needed treatments for HBM conditions by extending knowledge of the disease model to sites of greatest pathobiological relevance. The identification of robust otosclerosis also allows this model to be used when investigating this common disorder, highlighting the suitability of this preclinical model to widen the scope of pharmaceutical testing.

## MATERIALS AND METHODS

### Animals

Frozen *Sost^−/−^* sperm was purchased from the Knockout Mouse Project Repository at the University of California Davis, and used to fertilize ova from C57BL6 wild-type mice as described previously (Mosey et al., 2017). Male and female *Sost*^−/−^ mouse cohorts were generated by *Sost*^−/−^×*Sost*^−/−^ breeding, while C57BL6 mice were raised through C57BL6×C57BL6 breeding. Heterozygosity/homozygosity was confirmed by genotyping. Mice were housed in polypropylene cages with environmental enrichment and a 12/12 h light/dark cycle at 21±2°C. Mice had access to RM1 standard diet (LBS Biotechnology, Horley, UK) and water *ad libitum*. At the age of 9 months, animals were culled by exposure to CO_2_, confirmed by cardiac puncture. All procedures were approved by the Royal Veterinary College (RVC) ethics board and performed according to Animal Research: Reporting of In Vivo Experiments (ARRIVE) guidelines, in fulfilment of the UK Animals (Scientific Procedures) Act 1986 (Percie du Sert et al., 2020).

### Sample preparations and microCT image acquisition

Samples were fixed in paraformaldehyde (PFA) for 48 h, washed with Dulbecco's phosphate-buffered saline (DPBS) solution and stored in 70% ethanol. Skulls were wrapped in non-PVC plastic film and scanned using a Skyscan 1172F (Bruker, Kontich, Belgium). X-ray projection images, taken with a 0.6° rotation step over 180° sample rotation, at 13.5 μm voxel size, were acquired (software version 1.5). The X-ray tube was operated at 80 kV, 124 μA, and a 0.5 mm aluminium filter was used. Camera exposure time was optimised for linear signal response and flat fields refreshed prior to scans. Projection images were reconstructed (NRecon version 1.7.4.6, Bruker) with the following settings: Smoothing: 2; Ring artefacts correction: 15. Beam hardening correction was 35%. Grayscale threshold global values were set consistently for all scans based on image and histogram inspection.

### Image preparation and analysis

Samples were realigned in DataViewer (version 1.5.4.0, Bruker) to ensure consistent orientation. Image segmentation and 2D/3D morphometric analyses were performed using CTAn (version 1.20.3.0, Bruker). For morphometric skull analysis (including mandible), tissue volume (TV), bone volume (BV), bone volume fraction (BV/TV) and bone thickness were measured. Bone surface (BS), bone surface fraction (BS/BV) and ossicle thickness were included for otic capsules. Middle ear otic capsule cortical parameters, i.e. tissue and bone area (T.Ar and B.Ar), bone area fraction (B.Ar/T.Ar) and capsule thickness, were measured. The perimeter and area were measured for the foramen magnum. For whole-bone analysis of parietal bone section and mouse mandible, we included bone thickness, B.Ar, T.Ar, B.Ar/T.Ar, BV, bone perimeter (B.Pm), tissue perimeter (T.Pm), bone perimeter/area fraction (B.Pm/B.Ar) and bone porosity.

### Mouse hearing response

Male and female *Sost*^−/−^ mice aged 4-9 months were exposed to a high-frequency (∼20 kHz) tone stimulus at 90 dB sound pressure level (dB SPL), originating from a click-box positioned 30 cm above a single animal in its home cage (Hardisty-Hughes et al., 2010). Mice were then observed and graded for reactions and the presence or absence of a ‘Preyer’ reflex (i.e. outer ear flicked backwards in a hearing mouse). Grading categories were as follows: 0=no reaction; 1=no Preyer reflex; 2=retarded reaction; 3=Preyer reflex; 4=strong reaction; 5=particularly strong reaction.

### Statistical analysis

Data comparison was performed with GraphPad Prism 10.4.2 (Boston, Massachusetts, USA). Two-way ANOVA with Šidák's multiple comparison test was used to determine statistically significant differences for two-group comparisons [datasets: BL6 versus *Sost*^−/−^ or male versus female mice; data are shown as the mean and upper and lower quartiles (mean±s.d. is shown in Tables S1-S3]. Whole-bone 2D analyses of parietal bone sections and mandibles were completed in R version 4.2.3, using a linear mixed-effects model to assess differences between male and female C57BL6 and *Sost*^−/−^ mice at each distinct percentile along the bone section length. Shapiro–Wilk test was performed to assess normality of residuals; normality was assumed when most measures (10-90%) along the parietal bone section or mandible satisfied the null hypothesis of normality. Data are shown as the mean±s.e.m. and heatmaps that provide statistical significance (*P*<0.001; 0.001≤*P*<0.01; 0.01≤*P*<0.05 and *P*≥0.05) at each location were created. A *P*-value of ≤0.05 was used as the criterium for statistical significance.

## Supplementary Material

10.1242/dmm.052839_sup1 Supplementary information
