# Supplementary material for: Characterising skull pathologies in the Sost-deficient mouse for preclinical evaluation of sclerosteosis treatments
Source: Dis Model Mech. 2026 Aug 26;19(8):dmm052839. doi: 10.1242/dmm.052839 (PMC13580565; doi:10.1242/dmm.052839)
Supplement: Supplementary file 1 [file dmm-19-052839-s1.pdf]

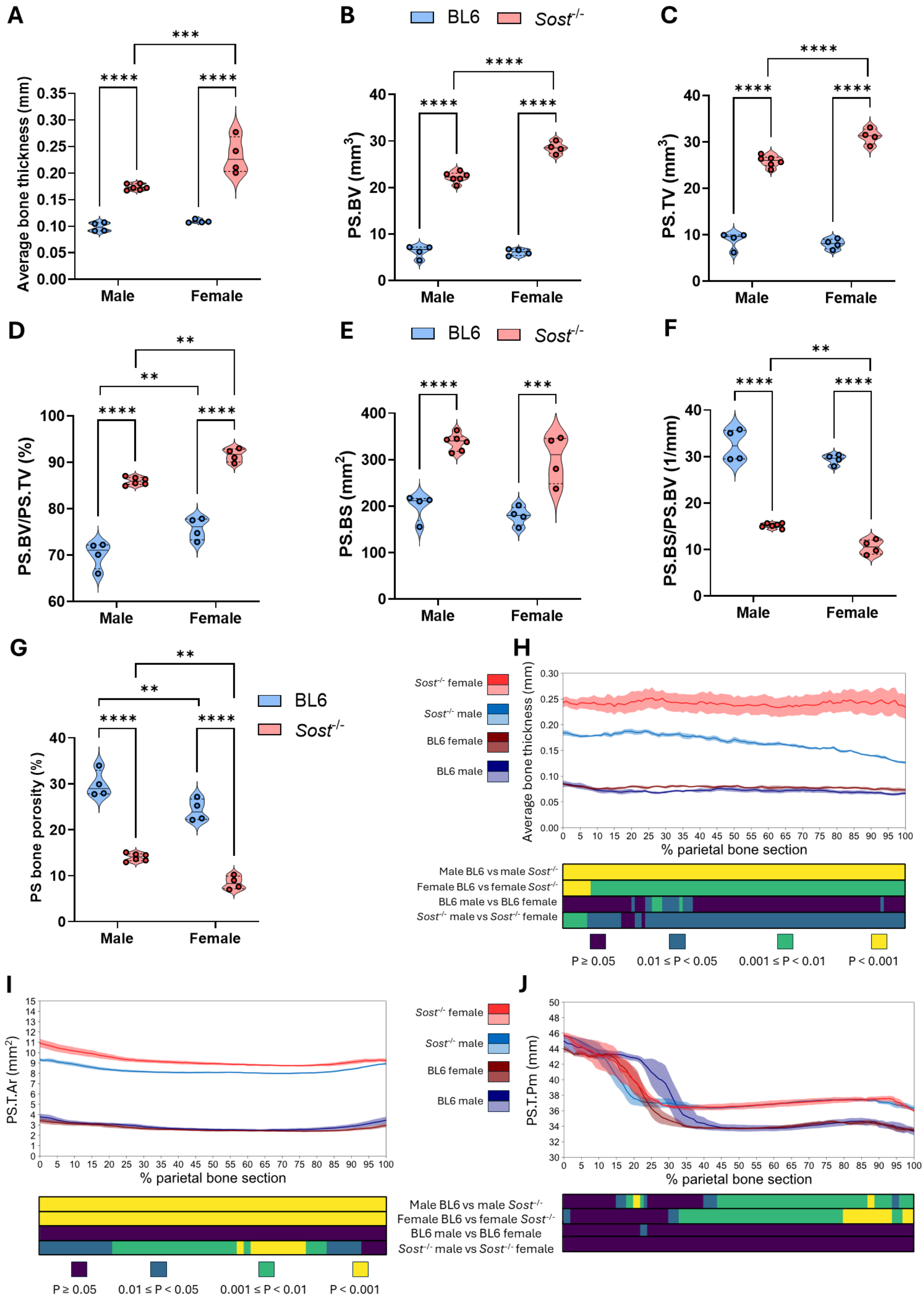

Fig. S1. Hyperostosis in a parietal/squamosal bone section in male and female *Sost*$^{-/-}$ mice. **A** Average parietal/squamosal bone thickness, (**B**) bone (PS.BV) and (**C**) total (PS.TV) volume, (**D**) percentage bone (PS.BV/PS.TV), (**E**) bone surface (PS.BS), (**F**)

Disease Models & Mechanisms • Supplementary information

bone surface fraction (PS.BS/PS.BV) and (**G**) porosity (PS bone porosity) of examined parietal/squamosal bone portion in male and female BL6 and *Sost*$^{-/-}$ mice. **H** Average bone thickness, (**I**) total parietal/squamosal area (PS.T.Ar) and (**J**) perimeter (PS.T.Pm) across whole parietal/squamosal bone regions. For graphs (**A** to **G**), means indicated by solid line, and upper and lower dashed lines represent quartiles. Blue and red represent BL6 and *Sost*$^{-/-}$ groups, respectively. $^{**}P \leq 0.01$, $^{***}P \leq 0.001$ and $^{****}P \leq 0.0001$. Line graphs (**H** to **J**) represent means ± SEM (BL6 males (dark blue); *Sost*$^{-/-}$ males (light blue); BL6 females (maroon); *Sost*$^{-/-}$ females (red)). Heat maps below graphs summarise statistical differences of above groups at matched locations along the analysed parietal/squamosal bone length (dark blue $P \geq 0.05$, blue $0.01 \leq P < 0.05$, green $0.001 \leq P < 0.01$, yellow $P < 0.001$). $n = 4$ BL6 males, $n = 4$ BL6 females, $n = 6$ *Sost*$^{-/-}$ males and $n = 4$ *Sost*$^{-/-}$ females.

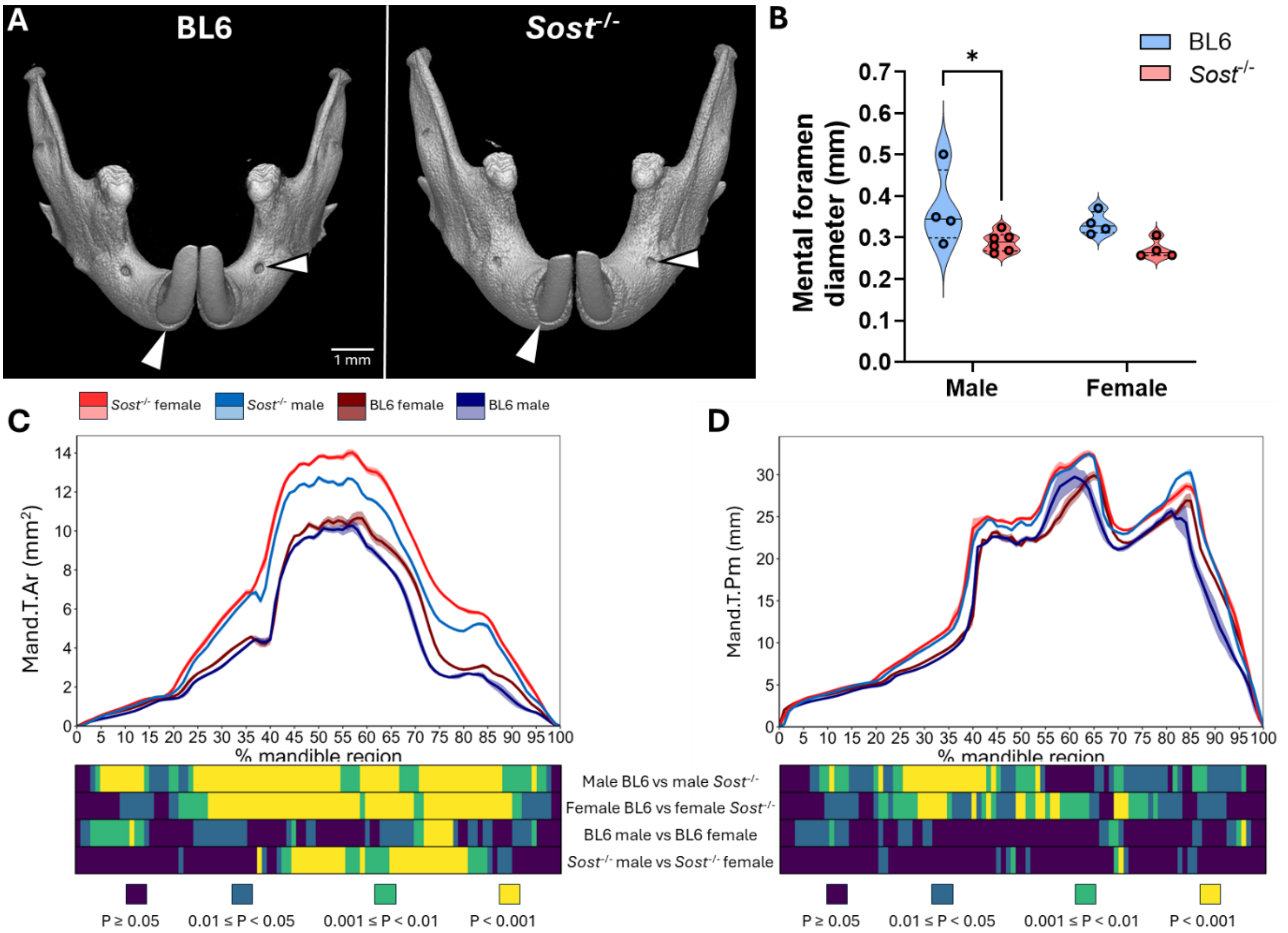

Fig. S2. Mandibular hyperostosis in male and female *Sost$^{-/-}$* mice. **(A)** 3D representative images from a female *Sost$^{-/-}$* mouse that highlights the mental foramen and bone overgrowth at the incisor base (white arrows). **(B)** Diameter of the BL6 and *Sost$^{-/-}$* mouse mental foramen. Means of each group are indicated by the solid line, and upper and lower dashed lines represent quartiles. Blue and red represent BL6 and *Sost$^{-/-}$* groups, respectively. **P* ≤ 0.05. **(C)** Total mandibular bone area (Mand.T.Ar) and (D) perimeter (Mand.T.Pm) across whole male and female BL6 and *Sost$^{-/-}$* mice mandibles Line graphs represent means ± SEM (BL6 males (dark blue); *Sost$^{-/-}$* males (light blue); BL6 females (maroon); *Sost$^{-/-}$* females (red)). Graphical heat maps below line graphs summarise statistical differences of the above groups at specific matched locations along the analysed length of the parietal bone section (dark blue *P* ≥ 0.05, blue 0.01 ≤ P < 0.05, green 0.001 ≤ *P* < 0.01, yellow *P* < 0.001). Group sizes were n =4 BL6 males, n = 4 BL6 females, n = 6 *Sost$^{-/-}$* males and n = 4 *Sost$^{-/-}$* females.

Disease Models & Mechanisms • Supplementary information

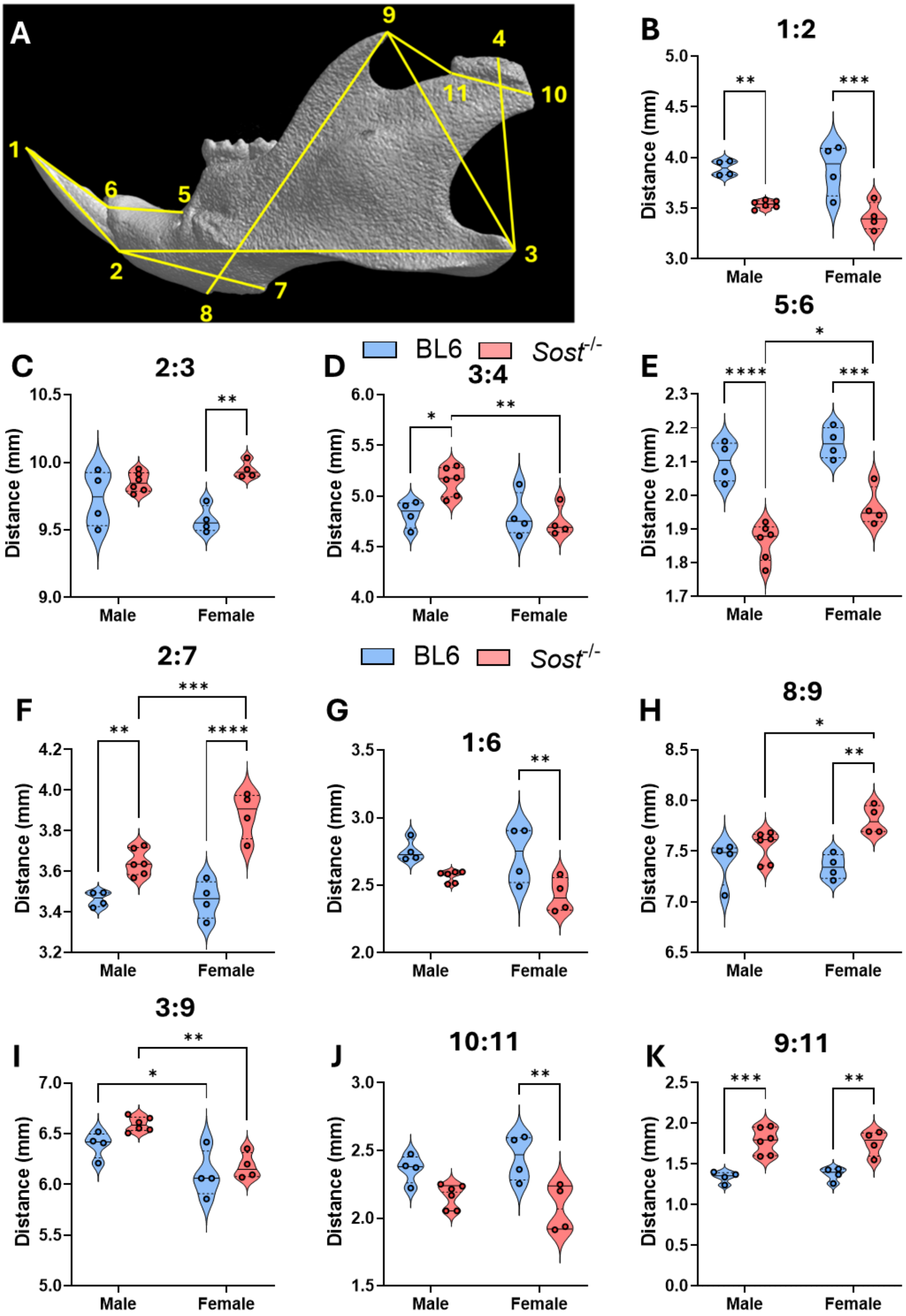

Fig. S3. Anatomical differences between male and female BL6 and *Sost$^{-/-}$* mandibles. (A) Selected landmarks and distances measured on the lateral side of the mandible. Distances measured (in mm) were from **(B)** landmark 1 to 2, **(C)** 2 to 3, **(D)** 3 to 4, **(E)** 5 to 6, **(F)** 2 to 7, **(G)** 1 to 6, **(H)** 8 to 9), **(I)** 3 to 9, **(J)** 10 to 11 and **(K)** 9 to 11 for male and female BL6 and *Sost$^{-/-}$* mice. Means indicated by solid line, and upper and lower dashed lines represent quartiles. Blue and red represent BL6 and *Sost$^{-/-}$* groups, respectively. $*P \leq 0.05$, $**P \leq 0.01$, $***P \leq 0.001$ and $****P \leq 0.0001$. Group sizes were n = 4 BL6 males, n = 4 BL6 females, n = 6 *Sost$^{-/-}$* males and n = 4 *Sost$^{-/-}$* females.

Disease Models & Mechanisms • Supplementary information

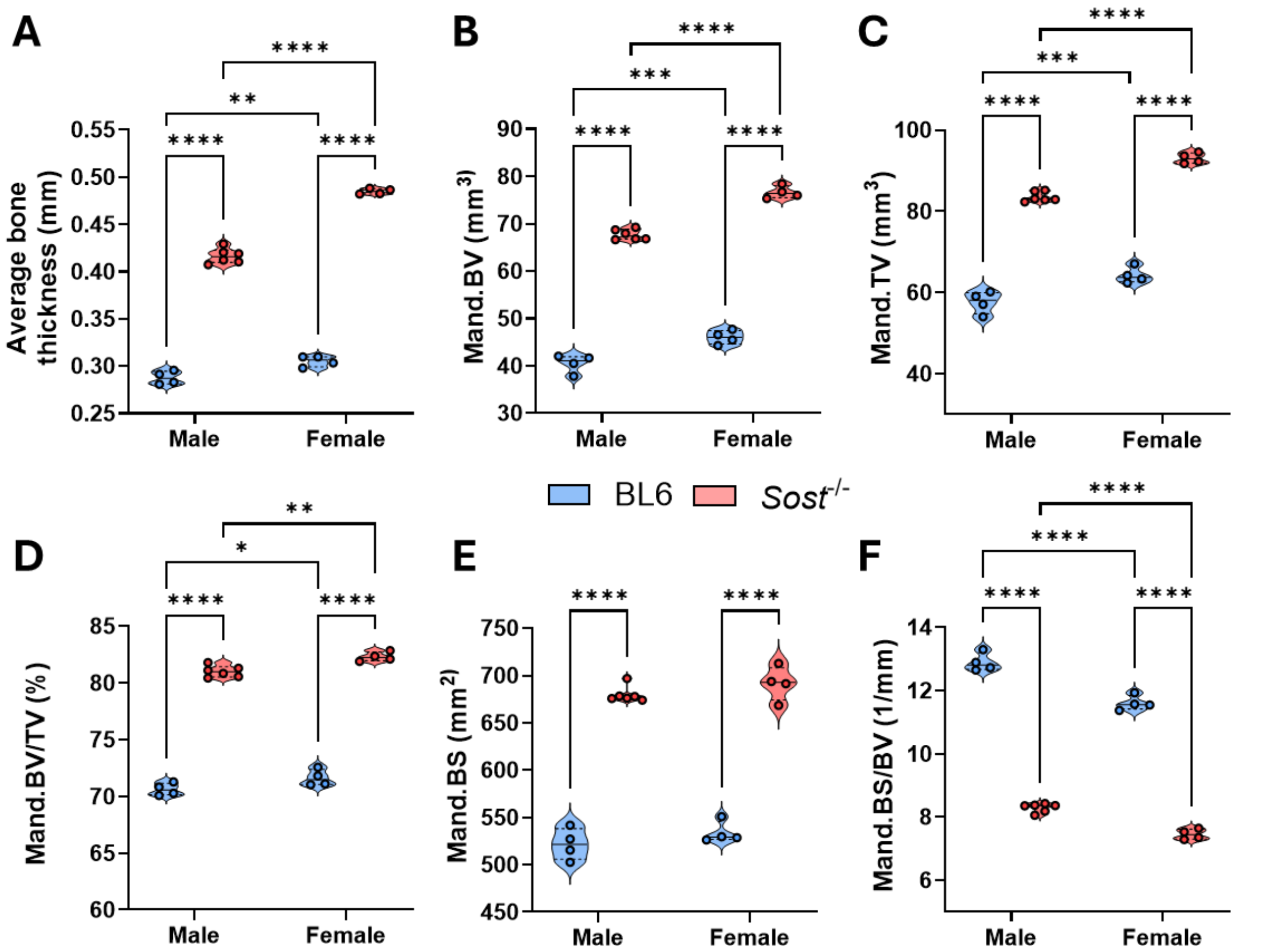

Fig. S4. Hyperostosis in male and female *Sost*$^{-/-}$ mandibles. Mandibular (**A**) average bone thickness, (**B**) bone volume (Mand.BV), (**C**) total volume (Mand.TV), (**D**) bone fraction (Mand.BV/Mand.TV), (**E**) bone surface (Mand.BS) and (**F**) bone surface fraction (Mand.BS/Mand.BV) of male and female BL6 and *Sost*$^{-/-}$ mice. Means are indicated by the solid line, and upper and lower dashed lines represent quartiles. Blue and red represent BL6 and *Sost*$^{-/-}$ groups, respectively. $^{**}P \leq 0.01$, $^{***}P \leq 0.001$ and $^{****}P \leq 0.0001$. $n = 4$ BL6 males, $n = 4$ BL6 females, $n = 6$ *Sost*$^{-/-}$ males and $n = 4$ *Sost*$^{-/-}$ females.

Disease Models & Mechanisms • Supplementary information

Table S1. Male and female $Sost^{-/-}$ and BL6 mouse bone morphometric parameters, comparing differences in genotypes and sex

*$P \leq 0.05$, **$P \leq 0.01$, ***$P \leq 0.001$ and ****$P \leq 0.0001$

Parameters	Sex	Genotype	Mean	± SD	Difference (%) between genotype for each sex	P-value	Genotype	Difference (%) between sex (female vs male) for each genotype	P-value
**Parietal bone**									
PS.BV (mm3)	Male	BL6	6.22	1.33	258	****	BL6	-2	
		$Sost^{-/-}$	22.24	1.12					
	Female	BL6	6.09	0.70	369	****	$Sost^{-/-}$	28	****
		$Sost^{-/-}$	28.56	1.28					
PS.TV (mm3)	Male	BL6	8.86	1.80	192	****	BL6	-9	
		$Sost^{-/-}$	25.86	1.18					
	Female	BL6	8.06	1.07	287	****	$Sost^{-/-}$	21	****
		$Sost^{-/-}$	31.21	1.67					
PS.BV/TV (%)	Male	BL6	70.09	2.88	23	****	BL6	8	**
		$Sost^{-/-}$	85.97	0.84					
	Female	BL6	75.74	2.37	21	****	$Sost^{-/-}$	7	**
		$Sost^{-/-}$	91.55	1.45					
PS.TS (mm2)	Male	BL6	129.89	16.81	14	*	BL6	-5	
		$Sost^{-/-}$	147.61	7.27					
	Female	BL6	123.01	9.40	32	***	$Sost^{-/-}$	10	
		$Sost^{-/-}$	162.55	6.25					
PS.BS (mm2)	Male	BL6	199.06	29.24	69	****	BL6	-10	
		$Sost^{-/-}$	337.20	18.12					
	Female	BL6	178.92	20.02	69	***	$Sost^{-/-}$	-11	
		$Sost^{-/-}$	301.64	52.27					
PS.BS/PS.BV (1/mm)	Male	BL6	32.50	3.42	-53	****	BL6	-9	
		$Sost^{-/-}$	15.17	0.50					
	Female	BL6	29.41	1.08	-64	****	$Sost^{-/-}$	-31	**
		$Sost^{-/-}$	10.53	1.56					
Average bone thickness (mm)	Male	BL6	0.10	0.01	76	****	BL6	11	
		$Sost^{-/-}$	0.17	0.01					
	Female	BL6	0.11	0.00	113	****	$Sost^{-/-}$	34	***
		$Sost^{-/-}$	0.23	0.03					
PS bone porosity (%)	Male	BL6	29.91	2.88	-53	****	BL6	-19	**
		$Sost^{-/-}$	14.03	0.84					
	Female	BL6	24.26	2.37	-65	****	$Sost^{-/-}$	-40	**
		$Sost^{-/-}$	8.45	1.45					
Intracranial	Male	BL6	377.01	14.19	-1		BL6	2	

		Sost$^{-/-}$	375.01	5.83					
	Female	BL6	385.95	5.31	-4	*	*Sost*$^{-/-}$	-2	
		Sost$^{-/-}$	369.35	7.71					
**Foramen magnum**									
Area (mm2)	Male	BL6	15.22	0.49	-11	****	BL6	3	
		Sost$^{-/-}$	13.48	0.14					
	Female	BL6	15.61	0.20	-17	****	*Sost*$^{-/-}$	-4	*
		Sost$^{-/-}$	12.96	0.25					
Perimeter (mm)	Male	BL6	15.17	0.16	-5	****	BL6	2	**
		Sost$^{-/-}$	14.41	0.05					
	Female	BL6	15.48	0.11	-9	****	*Sost*$^{-/-}$	-2	**
		Sost$^{-/-}$	14.13	0.13					
**Ossicles**									
Os.BV (mm3)	Male	BL6	0.16	0.00	32	****	BL6	10	**
		Sost$^{-/-}$	0.21	0.01					
	Female	BL6	0.18	0.01	18	****	*Sost*$^{-/-}$	-2	
		Sost$^{-/-}$	0.21	0.01					
Os.BS (mm2)	Male	BL6	3.97	0.10	38	****	BL6	10	**
		Sost$^{-/-}$	5.50	0.14					
	Female	BL6	4.38	0.15	27	****	*Sost*$^{-/-}$	1	
		Sost$^{-/-}$	5.58	0.15					
Os.BS/Os.BV (1/mm)	Male	BL6	24.52	0.09	5	*	BL6	0	
		Sost$^{-/-}$	25.70	0.39					
	Female	BL6	24.52	0.68	8	**	*Sost*$^{-/-}$	3	
		Sost$^{-/-}$	26.49	1.13					
Average ossicle thickness (mm)	Male	BL6	0.22	0.01	-7	**	BL6	0	
		Sost$^{-/-}$	0.20	0.01					
	Female	BL6	0.22	0.01	-8	**	*Sost*$^{-/-}$	-1	
		Sost$^{-/-}$	0.20	0.01					
**Otic capsule**									
Ot.B.Ar (mm2)	Male	BL6	0.76	0.09	109	****	BL6	8	
		Sost$^{-/-}$	1.58	0.13					
	Female	BL6	0.82	0.04	119	****	*Sost*$^{-/-}$	13	**
		Sost$^{-/-}$	1.79	0.04					
Ot.T.Ar (mm2)	Male	BL6	2.35	0.09	37	****	BL6	3	
		Sost$^{-/-}$	3.21	0.16					
	Female	BL6	2.43	0.03	38	****	*Sost*$^{-/-}$	4	
		Sost$^{-/-}$	3.35	0.10					
Ot.B.Ar/ Ot.T.Ar (%)	Male	BL6	32.12	2.71	53	****	BL6	5	
		Sost$^{-/-}$	49.07	1.78					
	Female	BL6	33.67	1.63	58	****	*Sost*$^{-/-}$	9	**
		Sost$^{-/-}$	53.29	0.42					
Average otic	Male	BL6	0.02	0.00	9	****	BL6	2	

Disease Models & Mechanisms • Supplementary information

		Sost$^{-/-}$	0.02	0.00					
	Female	BL6	0.02	0.00	8	****	*Sost*$^{-/-}$	1	
		Sost$^{-/-}$	0.02	0.00					
**Mouse hearing**									
Hearing reaction (4-5-month-old mice)	Male	BL6	4.75	0.50	-50	****	BL6	-23	*
		Sost$^{-/-}$	2.38	0.74					
	Female	BL6	3.67	0.50	-14		*Sost*$^{-/-}$	32	
		Sost$^{-/-}$	3.14	0.69					
Hearing reaction (8-9-month-old mice)	Male	BL6	2.83	0.41	-85	****	BL6	-9	
		Sost$^{-/-}$	0.43	0.53					
	Female	BL6	2.57	0.53	-34	**	*Sost*$^{-/-}$	297	***
		Sost$^{-/-}$	1.70	0.67					
**Mandible**									
Mand.BV (mm3)	Male	BL6	40.50	1.91	67	****	BL6	14	***
		Sost$^{-/-}$	67.71	1.13					
	Female	BL6	46.02	1.46	67	****	*Sost*$^{-/-}$	13	****
		Sost$^{-/-}$	76.63	1.33					
Mand.TV (mm3)	Male	BL6	57.60	2.72	45	****	BL6	12	***
		Sost$^{-/-}$	83.58	1.22					
	Female	BL6	64.25	2.03	45	****	*Sost*$^{-/-}$	11	****
		Sost$^{-/-}$	93.09	1.32					
Mand.BV/ Mand.TV (%)	Male	BL6	70.62	0.54	15	****	BL6	1	*
		Sost$^{-/-}$	81.01	0.51					
	Female	BL6	71.63	0.72	15	****	*Sost*$^{-/-}$	2	**
		Sost$^{-/-}$	82.31	0.41					
Mand.TS (mm2)	Male	BL6	292.05	9.98	0		BL6	-13	****
		Sost$^{-/-}$	291.14	2.98					
	Female	BL6	253.73	5.58	17	****	*Sost*$^{-/-}$	2	
		Sost$^{-/-}$	295.75	6.40					
Mand.BS (mm2)	Male	BL6	521.79	16.68	30	****	BL6	2	
		Sost$^{-/-}$	679.91	8.44					
	Female	BL6	533.86	11.41	30	****	*Sost*$^{-/-}$	2	
		Sost$^{-/-}$	691.71	18.14					
Mand.BS/ Mand.BV (1/mm)	Male	BL6	12.89	0.29	-36	****	BL6	-10	****
		Sost$^{-/-}$	8.29	0.14					
	Female	BL6	11.61	0.23	-36	****	*Sost*$^{-/-}$	-10	****
		Sost$^{-/-}$	7.45	0.16					
Average mandible thickness (mm)	Male	BL6	0.29	0.01	45	****	BL6	6	**
		Sost$^{-/-}$	0.42	0.01					
	Female	BL6	0.31	0.01	59	****	*Sost*$^{-/-}$	16	****
		Sost$^{-/-}$	0.48	0.00					
Mental forame	Male	BL6	0.37	0.09	-22	*	BL6	-10	

Disease Models & Mechanisms • Supplementary information

		Sost$^{-/-}$	0.29	0.02					
	Female	BL6	0.33	0.03	-18		*Sost*$^{-/-}$	-6	
		Sost$^{-/-}$	0.27	0.02					

Table S2. Sclerosteosis human patient and carrier data

	**Sex**	**Location**	**Mean**	**SD**	**Percentage change (%)**
**Patients (homozygous)** (Gardner *et al.*, 2005)					
Age	Male		30.40	16.77	45
	Female		44.00	14.14	
Z-score	Male	Spine	9.80	1.33	14
	Female		11.20	4.57	
	Male	Hip	9.35	1.31	11
	Female		10.36	1.63	
	Male	Forearm	6.48	1.72	23
	Female		7.97	2.21	
Age (Beighton *et al.*, 1976)	Male		28.17	16.09	-24
	Female		21.31	11.20	
Disease severity (tallied '+' symbols in Table 1) (Beighton *et al.*, 1976)	Male		5.83	1.95	-5
	Female		5.54	2.33	
**Carriers (heterozygous)** (Gardner *et al.*, 2005)					
Age	Male		33.00	20.06	45
	Female		47.78	17.09	
Z-score	Male	Spine	1.24	0.61	151 **
	Female		3.10	1.36	
	Male	Hip	1.14	0.69	32
	Female		1.51	1.00	
	Male	Forearm	1.24	1.07	39
	Female		1.72	0.72	

**$P \leq 0.01$

Disease Models & Mechanisms • Supplementary information

Table S3. Sclerosteosis human patient hearing data (Potgieter*et al.*, 2014)

	Sex	Side	Mean	SD	Percentage difference (%)
Age	Male		22.25	15.71	73
	Female		38.50	21.42	
Ear canal volume (ECV)	Male	Left	2.00	0.32	-20
	Female		1.60	0.54	
	Male	Right	2.45	0.96	-54
	Female		1.12	0.45	
Pure tone audiometry (PTA)	Male	Left	82.75	9.67	-11
	Female		74.00	20.18	
	Male	Right	76.00	14.31	5
	Female		79.83	11.27	
Speech recognition threshold (SRT) score	Male	Left	78.75	16.52	-4
	Female		75.83	20.84	
	Male	Right	75.00	11.55	7
	Female		80.00	16.43	
Mean Air Bone Gap (dB)	Male	Left	39.40	15.09	-39
	Female		24.12	11.49	
	Male	Right	31.38	10.57	-11
	Female		27.88	20.25	

References

Beighton, P., Durr, L. & Hamersma, H. 1976. The clinical features of sclerosteosis. A review of the manifestations in twenty-five affected individuals. *Annals of internal medicine,* 84**,** 393-397. (10.7326/0003-4819-84-4-393)

Gardner, J. C., Van Bezooijen, R. L., Mervis, B., Hamdy, N. A., Löwik, C. W., Hamersma, H., Beighton, P. & Papapoulos, S. E. 2005. Bone mineral density in sclerosteosis; affected individuals and gene carriers. *J Clin Endocrinol Metab,* 90**,** 6392-5. (10.1210/jc.2005-1235)

Potgieter, J. M., Swanepoel, D. W., Heinze, B. M., Hofmeyr, L. M., Burger, A. a. S. & Hamersma, H. 2014. An auditory profile of sclerosteosis. *The Journal of laryngology and otology***,** 1-9. (10.1017/s0022215113002648)

Disease Models & Mechanisms • Supplementary information
